# The toxic tango: TKI and ICI cardiotoxicities

**DOI:** 10.1186/s40959-022-00152-z

**Published:** 2023-12-06

**Authors:** Juan Del Cid Fratti, Vijayasree Paleru, Madhuri Bajaj, Chetan Bhardwaj

**Affiliations:** 1 Cardiology Department, OSF Healthcare/ University of Illinois at Peoria, Peoria, IL USA; 2 Oncology Department, OSF Healthcare/ University of Illinois at Peoria, Peoria, IL USA

**Keywords:** Cardiomyopathy, Myocarditis, ICI toxicity, TKI toxicity, ATE

## Abstract

**Background:**

Immune checkpoint inhibitors (ICI) and Tyrosine kinase inhibitors (TKI) are effective for several types of cancers, but they can have several cardiotoxicity sides effects. We present a case of TKI-ICI toxicity resulting in multiorgan inflammatory syndrome with myocarditis and thrombotic STEMI that were successfully treated with high-dose steroids and PCI.

**Case presentation:**

Seventy-two year-old man patient treated with on pembrolizumab 200 mg IV every 3 weeks and Axitinib 5 mg PO q12h for the past 5 months complained of acute shortness of breath, altered mental status, and chronic diarrhea. Coronary angiography demonstrated a thrombotic lesion in the right coronary artery (RCA) that was treated successfully with percutaneous coronary intervention (PCI). Despite PCI he continued to complain of shortness of breath further workup with Cardiac MRI (CMR) was obtained showed an ejection fraction of 38%, small pericardial effusion, and delayed gadolinium enhancement (DGE) in the inferior wall suggestive of myocarditis. An empirical trial of high-dose steroids improved all patient symptoms and ejection fraction; therefore, the chemotherapy regimen was changed.

**Conclusion:**

This case report highlights the potential vasculogenic effects of Axitinib and immune-related myocarditis of pembrolizumab. Cardiologists and oncologists should be vigilant for the cardiotoxic effects of Axitinib and pembrolizumab.

**Supplementary Information:**

The online version contains supplementary material available at 10.1186/s40959-022-00152-z.

## Background

Renal cell carcinoma is the most common form of renal cancer. Most patients present with localized disease that is amenable to definitive surgical treatment. However, 30% of patients have metastatic disease at their initial presentation. The introduction of targeted therapies such as tyrosine kinase inhibitors and immunotherapy with checkpoints inhibitors (ICI) have improved the medial survival [1]. Axitinib a vascular endothelial growth factor (VEGF) TKI and Pembrolizumab an ICI are effective for advanced RCC; however, is there are known to have vasculogenic effects and immune-related events including fulminant myocarditis [2, 3]. Hereby, we present a case of an axitinib-related thrombotic acute coronary syndrome (ACS) and pembrolizumab-related myocarditis confirmed with an excellent response by high-dose steroids.

## Case presentation

**Fig. 1:**
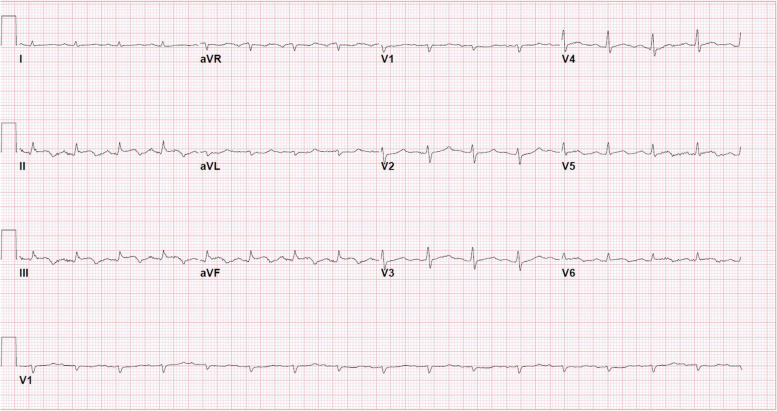
ECG with ST segment elevation in inferior leads

A 72-year-old man presented with acute shortness of breath. Telemetry showed ST elevation, but this was not evident in a 12-lead ECG. Before life-flight to our center shortness of breath worsen, and ECG showed ST elevation in inferior leads (Fig. 1). His vital signs were significant for a heart rate of 102 beats/min, blood pressure 140/80 mmHg, afebrile, and oxygen saturation of 90% on room air. Physical exam notable for confusion not able to provide a detailed history.

### Past medical history

His medical history was significant for CKD, dyslipidemia, and renal cell carcinoma, which was diagnosed 8 months before presentation, after a 12.8 cm right renal mass was found. 14-days later underwent radical right nephrectomy which showed clear cell renal cell carcinoma (RCC) limited to kidneys. Follow-up CT scan showed 4 cm inferior left adrenal gland mass avid for FDG-PET and IR guided biopsy demonstrated RCC. Was started on pembrolizumab 200 mg IV every 3 weeks and axitinib 5 mg PO q12h for the past 5 months.

### Investigation

**Fig. 2:**
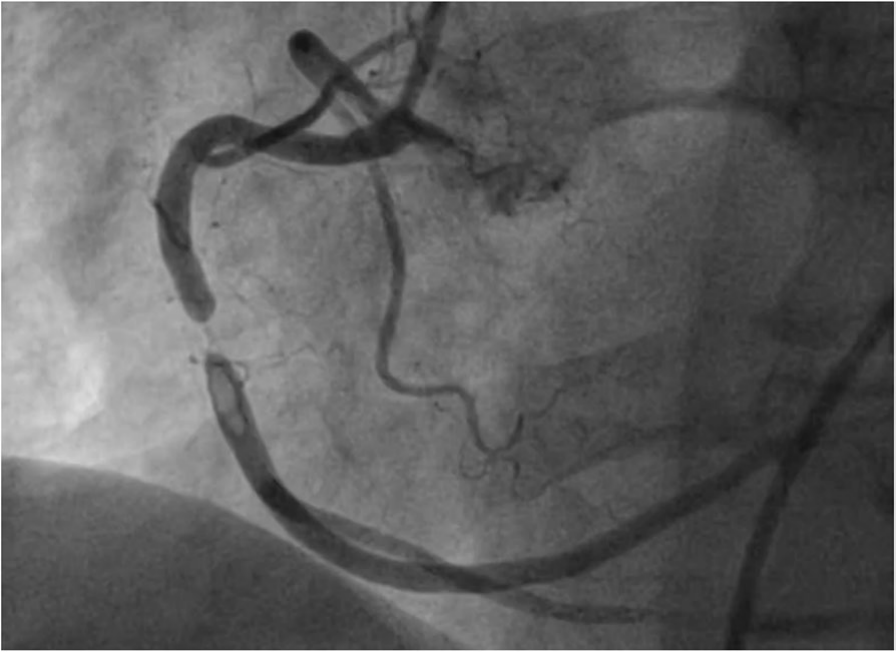
A thrombotic lesion in the mid-right coronary artery

**Fig. 3:**
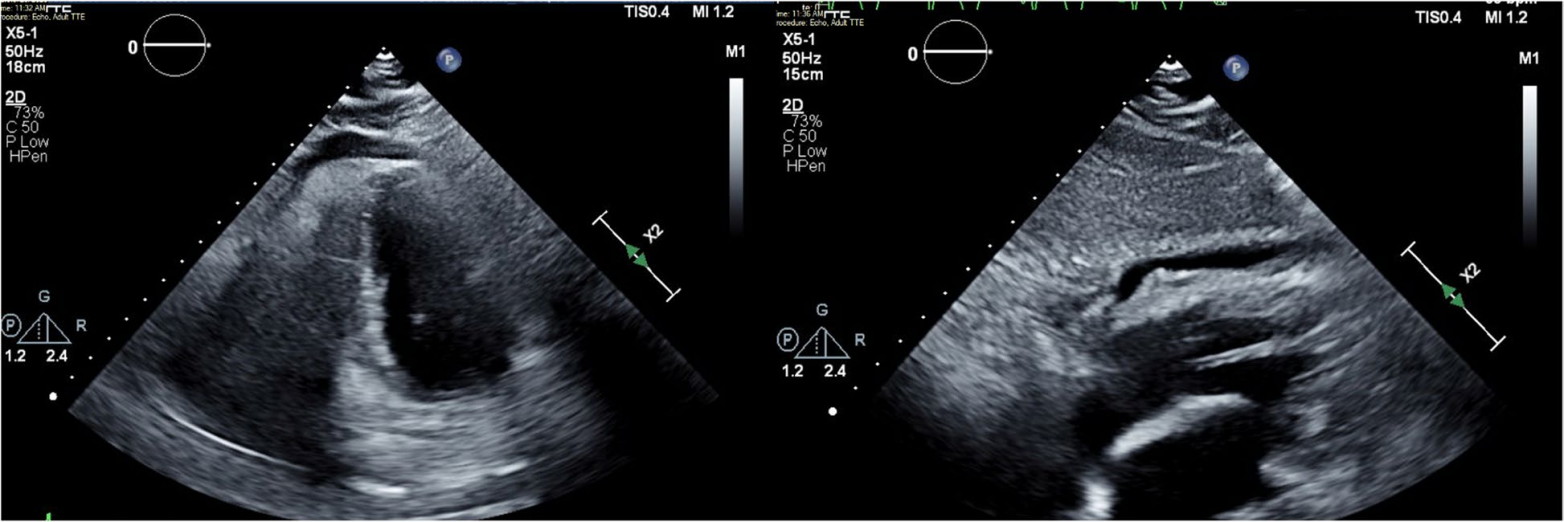
Echocardiogram with small to moderate pericardial effusion with organized material

**Fig. 4:**
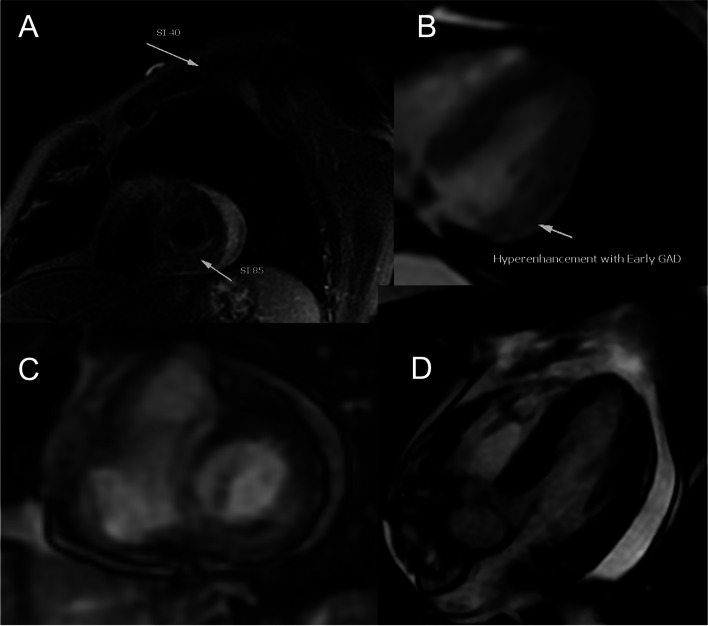
Cardiac MR showing myocardial edema

Coronary angiogram showed a thrombotic lesion in RCA with normal rest of coronaries without evidence of atherosclerosis or bystander plaque which underwent successful PCI with a DES (Fig. 2). After PCI patient provided more history, has been complaining of dyspnea on exertion for the past 3 months that is worsening, chronic diarrhea, and waxing, and waning altered mental status. Transthoracic echocardiogram showed inferior wall hypokinesis, normal ejection fraction, normal diastolic function, and small to moderate pericardial effusion with organized material (Fig. 3). CRP was elevated at 3 mg/mL and ESR at 42 cm. During the next days, shortness of breath did not improve, and the patient was not able to walk more than 20 feet and supplemental oxygen is needed. We perused further workup with VQ scan which showed a very low probability of pulmonary embolism, Pulmonary function test (PFT) with mild obstructive lung defect with severe reduction of diffusion capacity, high-resolution CT chest showed moderate emphysema, bronchial wall thickening and septal thickening, and normal hemodynamics in right heart catheterization (Fig. 4, Additional file 1: Fig. S1 and Additional file 2: Fig. S2).

### Differential diagnosis and further work-up

At this time patient is still complaining of severe shortness of breath with mild exertion, multiorgan involvement with chronic diarrhea, mild elevation of liver function test, pericardial effusion, altered mental status, and possible pneumonitis. Cardiac MRI was obtained showed moderate complex hemorrhagic pericardial effusion with internal debris with enhancement in FIESTA images, LVEF 38%, triple IR (T1W) showed increased signal intensity basal to mid inferior and inferior-lateral wall with signal intensity (SI) ratio > 2 compared to skeletal muscle, early GAD imaging (600 TI) showed hyperenhancement basal to mid inferolateral wall, and myocardial LGE imaging showed mid myocardium enhancement in basal to mid inferior and inferolateral wall. (Additional file 3: Fig. S3). The main differential was ICI-multiorgan inflammation with possible myocarditis due possible CMR findings (Based on prior Lake Louis Criteria) and myocarditis syndrome.

### Treatment and follow-up

An empirical trial of IV-methylprednisone 500 mg IV q12 was started with drastic improvement of shortness of breath, resolution of AMS, and discontinuation of oxygen. Discharge with prednisone taper, and GDMT for HF. At 2, and 6 weeks visit the dyspnea on exertion, diarrhea, and AMS diarrhea had resolved. Because of recent myocardial infarction we did not trend cardiac troponins to avoid confounders. Follow-up echocardiogram improvement of ejection fraction with the resolution of pericardial effusion and wall motions. Plan for multidisciplinary meeting for evaluation of further chemotherapy.

## Discussion

The age of the population in the United States (US) is increasing for the past decades, and there is an increase of long-term cancer survivors due to better treatment options leading to more elderly patients with cardiovascular disease and cancer [4]. There are several ICI approved by the FDA and their use is increasing over time. In this case, pembrolizumab was used for metastatic RCC but is also approved for other types of cancer [2, 5]. The most common irAES related to ICI reported are colitis, pneumonitis, hepatitis, hypophysitis, neurological, and adrenal. Myocarditis-related irAES is not common but is the irAES with the highest fatality rate [6]. ICI act on PD-1, CTLA-4, and PD-L1 receptors activating the T-cell leading to the killing of the tumor cells. Murine models have demonstrated that PD-L1 expression is also present on injured cardiomyocytes and for this reason activated T cells can lead to the destruction of these cells and lymphocytic myocarditis [7]. Due to the high mortality rate definitions of ICI myocarditis have been proposed with a definitive diagnosis with pathology results or classic CMR findings plus syndrome, probable if suggestive CMR findings are present with syndrome, and possible diagnosis myocarditis with suggestive CMR findings without syndrome, this is to ensure rapid recognition and early treatment [8]. The severity of myocarditis has been proposed from mild symptoms to severe with a life-threatening disease, and EF < 50% [2]. Cardiac MR findings suggestive of myocarditis had been defined we used Lake Louis Criteria from 2018 where the two major criteria are myocardial edema by T2 mapping, non-ischemic myocardial injury by T1, ECV or LGE, and supportive criteria (Pericarditis by presence of effusion in FIESTA, and systolic LV dysfunction or wall motion abnormalities). This Criteria had been updated with the use of parametric mapping but at the time of evaluation of the patient our institution did not have parametric mapping available [9].

Due to high complexity and morbidity, the treatment of immune checkpoint inhibitor myocarditis requires a team approach with a cardiologist and hematology-oncologist. After clinical suspicion is recommended to stop the ICI, perform work diagnostic work-up, and initiate high dose steroids. The American Society of Clinical Oncology Clinical practice guidelines recommend holding the ICI permanently for myocarditis with symptoms and high dose steroids with 1-2 mg/kg oral prednisone or IV depending on symptoms for all-grade toxicities [10]. While the American heart association gives a treatment algorithm with an aggressive strategy with 1000 mg IV methylprednisolone daily for 3 days or until clinical stability is achieved, and if diagnosis workup reveals definitive, probable, or possible myocarditis and there is no other explanation of clinician syndromes continuation of steroids with prednisone taper for 4–6 weeks or longer as per clinical and biomarker response is recommended. If there is no improvement with steroids other options can be considered like infliximab, IVIG, plasmapheresis, abatacept, and alemtuzumab [2]. Due to high mortality and data of recurrence [11], a repeated trial of ICI in patients with previous ICI-myocarditis is not recommended.

Axitinib was part of our patient chemotherapy regimen for advanced RCC [5], is a selective second-generation TKI that is recommended as adjunctive therapy with ICI for metastatic RCC. The most common concerning cardiovascular irAES reported are arterial thromboembolic events (ATE), and severe hypertension. Mechanism of these irAES id due imbalance in endothelium vasodilatory and vasoconstricting response, with profound vasospasm and platelet activation leading to thrombosis and hypertension [3]. In cases of new or acute ischemia as in our case therapy with TKI should be only resumed if the benefit outweighs the risk. As the numbers of cancer survivors and use of ICI increase, the number of cases of myocarditis is expected to increase, there is a need for a multidisciplinary approach for these patients’ involving oncology, and cardiology to improve survival and outcomes.

## Conclusion

ICI-Myocarditis is a severe irAE. The diagnosis requires clinical suspicion, and exclusion of other cardiac conditions due to the high fatality rate early recognition and prompt treatment are needed to improve mortality. TKI related arterial ATE is not common but if present different therapies options should be offered.

## Learning objectives

- TKI- related ATE is not common but should be suspected in patients with thrombotic coronary artery disease without bystander plaque or coronary artery disease.
- ICI-myocarditis has low incidence but carries a high mortality. Early recognition and treatment are needed.
- The cardio-Oncology team should be involved in patients undergoing complex chemotherapy agents.

### Supplementary Information

**Additional file 1: Fig. S1. **VQ Scan.

**Additional file 2: Fig. S2. **CT Scan.

**Additional file 3: Fig. S3. **PFT test.

## Data Availability

Upon request.

## Abbreviations

ICI: Immune checkpoint inhibitor
TKI: Tyrosine kinase inhibitor
VEGF: Vascular endothelial growth factor
STEMI: ST-elevation myocardial infarction
PCI: Percutaneous coronary intervention
DES: Drug eluting stent.
irAEs: Immune related adverse events
ATE: Arterial thromboembolism.
RCC: Renal cell carcinoma
HFrEF: Hear failure reduced ejection fraction
CMR: Cardiac magnetic resonance
