# Correction: The toxic tango: TKI and ICI cardiotoxicities

**DOI:** 10.1186/s40959-024-00200-w

**Published:** 2024-01-18

**Authors:** Juan Del Cid Fratti, Vijayasree Paleru, Madhuri Bajaj, Chetan Bhardwaj

**Affiliations:** 1 Cardiology Department, OSF Healthcare/ University of Illinois at Peoria, Peoria, IL USA; 2 Oncology Department, OSF Healthcare/ University of Illinois at Peoria, Peoria, IL USA

**Correction: Cardio-Oncology 9, 44 (2023)**

10.1186/s40959-022-00152-z

Following publication of the original article [1], the authors reported an error found in the article title and the second author’s given name.

Article title (incorrect):

The toxic tango: TKI and TCI cardiotoxicities

Article title (correct):

The toxic tango: TKI and ICI cardiotoxicities

Author name (incorrect):

Vijaysree Paleru

Author name (Correct):

Vijayasree Paleru

The original article [1] has been updated.
