# Supplementary figures and images for: The toxic tango: TKI and ICI cardiotoxicities
Source: Cardiooncology. 2023 Dec 6;9:44. doi: 10.1186/s40959-022-00152-z (PMC10698952; doi:10.1186/s40959-022-00152-z)

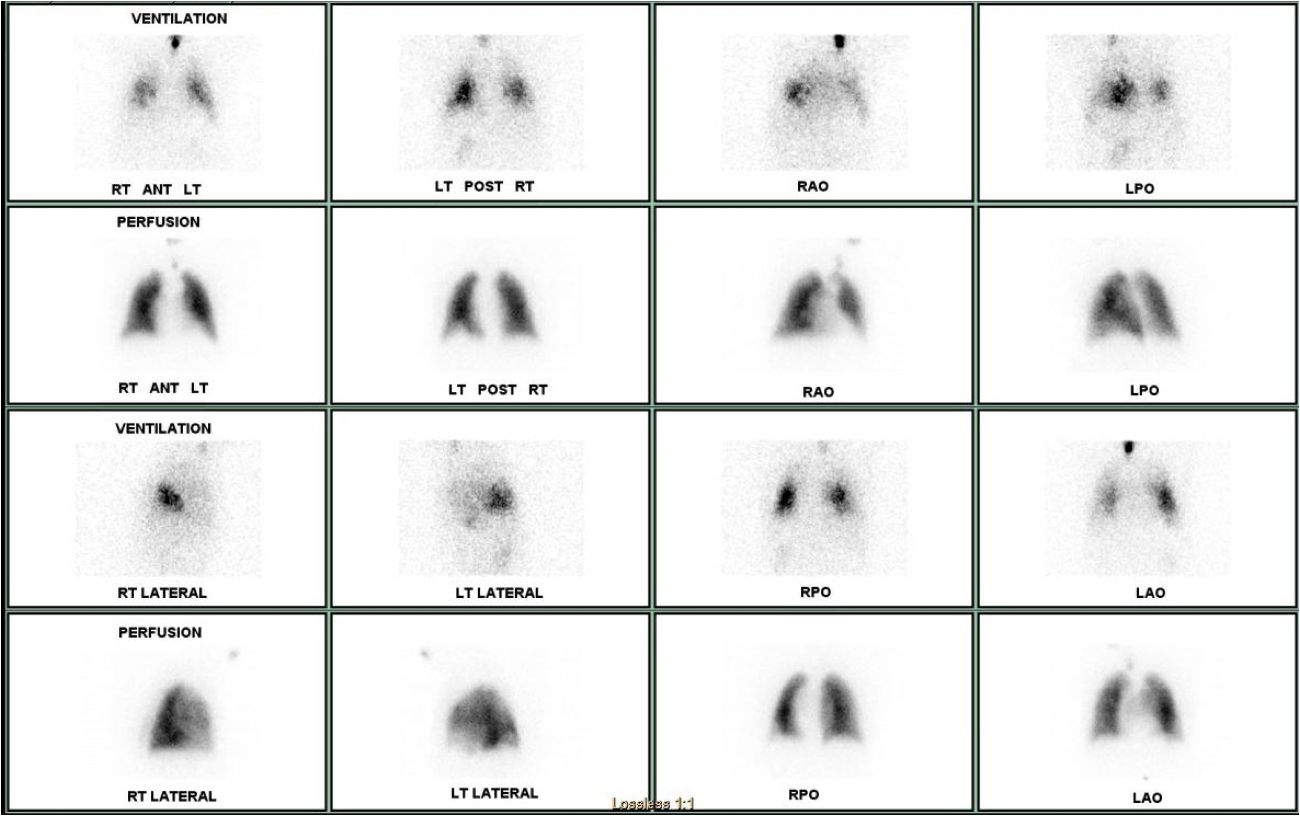

Supplement: Supplementary file 1 — Additional file 1: Fig. S1. VQ Scan. [file 40959_2022_152_MOESM1_ESM.png]

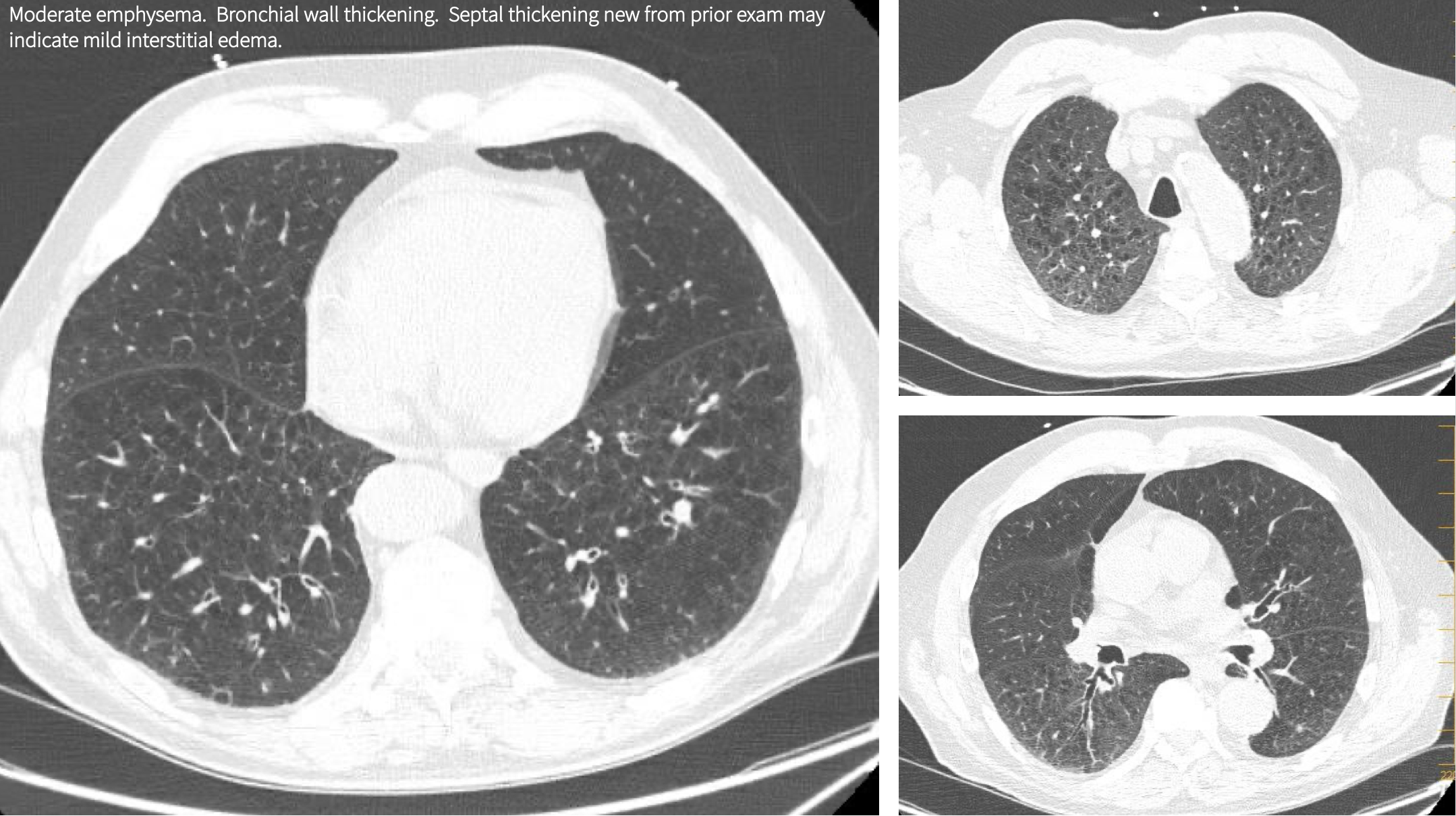

Supplement: Supplementary file 2 — Additional file 2: Fig. S2. CT Scan. [file 40959_2022_152_MOESM2_ESM.png]

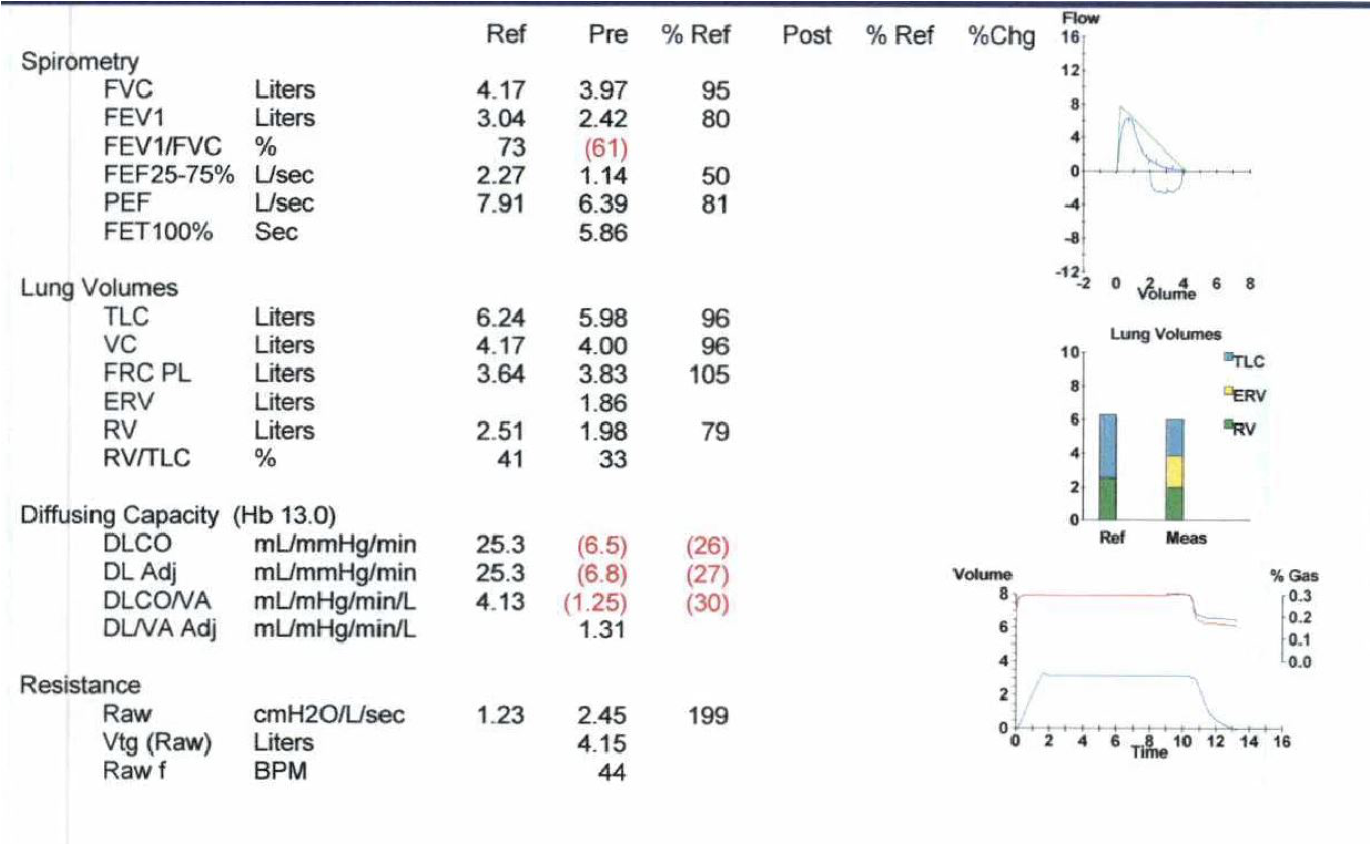

Supplement: Supplementary file 3 — Additional file 3: Fig. S3. PFT test. [file 40959_2022_152_MOESM3_ESM.png]
